# Changes in the Relative Abundance and Movement of Insect Pollinators During the Flowering Cycle of *Brassica rapa* Crops: Implications for Gene Flow

**DOI:** 10.1673/031.013.1301

**Published:** 2013-02-18

**Authors:** Laura A. Mesa, Bradley G. Howlett, Jan E. Grant, Raphael K. Didham

**Affiliations:** 1 School of Biological Sciences, University of Canterbury, P.O. Box 4800, Christchurch, New Zealand; 2 Sustainable Production, The New Zealand Institute for Plant and Food Research Limited, Private Bag 4704, Christchurch, New Zealand; 3 Breeding and Genomics, The New Zealand Institute for Plant and Food Research Limited, Private Bag 4704, Christchurch, New Zealand; 4 School of Animal Biology, The University of Western Australia, 35 Stirling Highway, Crawley WA 6009, Australia; 5 CSIRO Ecosystem Sciences, Centre for Environment and Life Sciences, Underwood Ave, Floreat WA 6014, Australia

**Keywords:** *Brassica*, flower phenology, insect pollination, pollinator dispersal, temporal variation

## Abstract

The potential movement of transgenes from genetically modified crops to non-genetically modified crops via insect-mediated pollen dispersal has been highlighted as one of the areas of greatest concern in regards to genetically modified crops. Pollen movement depends sensitively on spatial and temporal variation in the movement of insect pollinators between crop fields. This study tested the degree of variation in the diversity and relative abundance of flower-visiting insects entering versus leaving pak choi, *Brassica rapa* var. *chinensis* L. (Brassicales: Brassicaceae), crops throughout different stages of the flowering cycle. The relative abundance of flower-visiting insects varied significantly with *Brassica* crop phenology. Greater numbers of flower-visiting insects were captured inside rather than outside the crop fields, with the highest capture rates of flower-visitors coinciding with the peak of flowering in both spring-flowering and summer-flowering crops. Moreover, the ratio of flower-visiting insects entering versus leaving crop fields also varied considerably with changing crop phenology. Despite high variation in relative capture rates, the data strongly indicate non-random patterns of variation in insect movement in relation to crop phenology, with early-season aggregation of flower-visiting insects entering and remaining in the crop, and then mass emigration of flower-visiting insects leaving the crop late in the flowering season. Although pollen movement late in the flowering cycle might contribute relatively little to total seed set (and hence crop production), the findings here suggest that extensive late-season pollinator redistribution in the landscape could contribute disproportionately to long-distance gene movement between crops.

## Introduction

Insect-mediated pollen transfer is a key mechanism for gene movement and cross-pollination within and between a wide range of crops (Fenster 1991; Ellstrand and Elam 1993; Ennos 1994; Ghazoul 2005). Therefore, the abundance and diversity of the pollinator assemblage is not only important for seed production within crops (Suberi and Sarker 1992; Stewart 2002; Klein et al. 2007), but also for viable pollen movement between crops (Chifflet et al. 2011; Rader et al. 2011). This pollen movement between crops could result in unwanted cross-pollination of conventional crops, weeds, and wild relatives (Poppy and Wilkinson 2005; Hoyle et al. 2007). Interest in pollen movement from crops has increased with the mass-planting of genetically modified crops (Chifflet et al. 2011), due to the potential risk of uncontrolled ‘escape’ of introduced genes. Gene escape might be particularly problematic in massflowering crops that are attractive to a wide range of generalist flower-visiting species with differing relative abundances and dispersal capabilities (e.g., Abrol and Kapil 1996; Rader et al. 2011). In particular, it is crucial to understand how the abundance and distribution of these different flower-visiting species vary throughout the growing season (Howlett et al. 2005), and how the relative activity rates of flower-visitors entering and leaving mass-flowering crops vary with changing crop phenology, as these factors will have an important bearing on the relative risk of gene movement in the landscape.

Insect-mediated pollen transfer in massflowering *Brassica* crops has been particularly well studied, as insect pollinator activity can contribute significantly to pollination (Sihag 1985; Hayter and Cresswell 2006; Rader et al. 2009). A large number of insect species visit *Brassica* flowers (Howlett et al. 2009a, b, 2011), and absolute visitation rate is thought to play a central role in the resulting quality and yield of seed (Bhalla et al. 1983). For example, some authors have found that without adequate cross pollination, *Brassica rapa* var. *chinensis* L. (Brassicales: Brassicaceae) cannot produce high seed yield (Suberi and Sarker 1992; Westcott and Nelson 2001). Similarly, Morandin and Winston (2005) found that herbicide-driven reductions in pollinator abundance resulted in poor seed set in genetically modified *B. rapa* crops in Canada. To some extent, however, the effect of pollinator abundance on seed set is likely to be dependent on the cultivars planted, the environmental conditions where the crop grows, and the compensatory capacity of the crop (Mesquida et al. 1988; Free 1993; Westcott and Nelson 2001).

A wide variety of insects have been recorded as flower-visitor s in *Brassica* crops. These include social bees (Donovan 1980; Goodell and Thomson 2006), solitary bees (e.g., *Leioproctus* and *Lasioglossum*) (Rader et al. 2009; Howlett et al. 2009a; Rader et al. 2011), Diptera, Lepidoptera, and Coleoptera (Brunei et al. 1992; Chaudhary 2001; Howlett 2009a, b; Walker et al. 2009; Chifflet et al. 2011; Rader et al. 2011). Across all the taxa recorded as flower visitors of *Brassica* crops, *Bombus* spp. and *Apis mellifera* are considered key visitors in most regions of the world (Sihag 1986; Singh and Singh 1992; Williams 1997), and are typically considered to control the maximum rate of pollination (Hayter and Cresswell 2006). Therefore, variation in the abundance of social bees visiting flowers, either spatially or seasonally, might be expected to have a dominant influence on gene flow and cross-pollination within and among crop fields (DeGrandi-Hoffman et al. 1992; Firbank et al. 2003; Hayter and Cresswell 2006; Sahli and Conner 2007). However, recent studies have shown that many other solitary insects are also capable of transporting viable *Brassica* pollen over very large distances, and are significant contributors to the movement of pollen from crops (Chifflet et al. 2011; Rader et al. 2011).

Although studies such as Rader et al. (2011) and Chifflet et al. (2011) provide much sought-after evidence of the complexity of pollen movement by insects from *Brassica* crops, they provide limited insight into the influences of temporal factors, such as the seasonality of crop plantings, or changing crop phenology during the period of crop flowering. These factors may significantly influence pollen movement by insects, and hence influence gene flow. For example, gene flow between genetically modified and nongenetically modified crops was found to be twice as high in winter-sown crops, which bloomed in spring when pollinator abundance was low, compared to spring-sown crops, which bloomed in summer when pollinator diversity and abundance were high (Weekes et al. 2005). In contrast, we are unaware of studies that have extended observations of insect movement across the full flowering cycle in *Brassica* crops to examine how the relative activity rates of different flowervisiting species in mass-flowering crops differ with changing crop phenology. In the present study, the aim was to quantify the degree of spatial and temporal variation in the diversity and relative abundance of key flower-visiting species entering versus leaving pak choi (*B. rapa*) crops throughout the crop development and flowering cycle in an intensive cropping landscape in New Zealand.

## Materials and Methods

Crop phenology and insect activity patterns were measured in two 50 × 50 m fields of *B. rapa* located at Lincoln on the Canterbury Plains, South Island, New Zealand. The soil in both fields was a Wakanui Silt Loam. *Brassica* seeds were drilled to 2 cm depth at 15 cm square spacing on 12 September 2006 (Lincoln ‘spring-flowering’ crop) and 10 November 2006 (Lincoln ‘summer-flowering’ crop). The fields were 3 km apart. The amount of seed sown was 200 kg seeds ha-1. Weeds were controlled with Trifluran at 1.7 L ha-1. Fertilizer was applied according to common practices used at Plant and Food Research Ltd. before planting (the details of rate of application and fertilizer composition are commercially sensitive, and are therefore not available for release).

### Crop phenology

Crop phenology was monitored at weekly intervals, from the time the first leaves appeared on the emerging seedlings until seeds were formed on mature plants, in both crops. On each sampling date, plant and flower density measurements were recorded within three randomly-located 1-m^2^ quadrats at the center, and at each of the four corners of the crop (giving a total of 15 samples per week). The number of plants in each 1-m^2^ sample was recorded, and the number of inflorescences per plant was estimated by counting the inflorescences on 10 randomly-selected plants. The numbers of buds, open flowers, and old (senescent) flowers per inflorescence were estimated by counting the numbers of each flower type in 10 randomly selected inflorescences within each of the 10 plants. These variables were recorded weekly until the flowering phase had ceased.

### Abundance and diversity of flower-visiting insects

**Figure 1.**
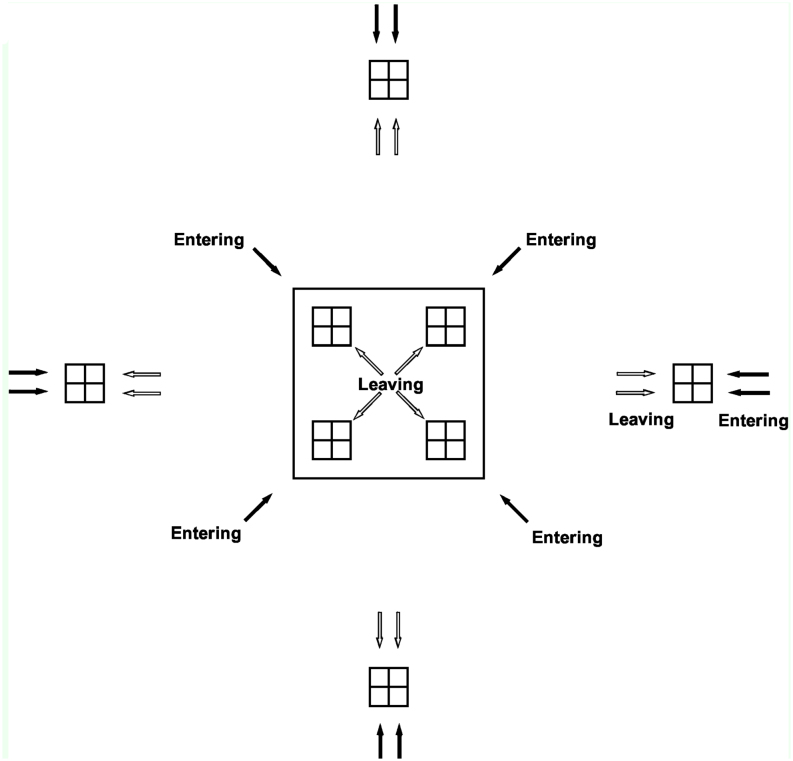
Diagram of one of the two *Brassica rapa* var. *chinensis* crop fields showing the locations of four window traps inside the crop, each with one quadrant designated for the capture of insects entering the field, and the diagonally-opposite corner for the capture of insects leaving the field, as well as the locations of four window traps outside the crop (50 m from the edge of the field). High quality figures are available online.

The relative activity rates of flower-visiting insects were estimated using four flight intercept (window) traps located inside the crop in each of the four corners (5 m from the crop edge), and four window traps located outside the crop at 50 m distance from the crop edge (Figure 1). Capture rates in the type of window traps used in this study have been shown to be highly correlated with observed visitation rates at flowers (Howlett et al. 2009a).

Each trap consisted of a rectangular grey 6 L plastic tray that supported two transparent Perspex (acrylic) window panes intersecting perpendicularly. The Perspex pane that ran lengthways along the tray had dimensions of 36.4 cm wide by 27.0 cm high (tapering at the tray base to 34.7 cm), while the pane running perpendicular to the tray was 23.8 cm wide by 27.0 cm high (tapering to 21.8 cm wide at the tray base). Four long stakes (1.2 m aluminium coated with green plastic) were hammered into the ground at a height that was just below the height of the crop flowers, in a pattern that matched the trap dimensions. The grey plastic tray was attached to the stakes using 15 cm long copper tubing to connect the stakes with the plastic tray joiners (Howlett et al. 2009a). The window trap was then placed on top to ensure that it was positioned at the same height as the flowers.

Window traps were oriented with the longest side pointing north. For the traps inside the crop, the exterior-most diagonal corner of each trap was designated as capturing insects entering the crop, while the interior-most diagonal corner of each trap was designated as capturing insects leaving the crop, as depicted in Figure 1. Insect samples from the other two quadrants of each trap were not analyzed here because it was much less likely that these could be interpreted as insects entering or leaving the crop with any great certainty. For the four traps outside the crop, the two quadrants of each trap that were closest to the crop were designated as capturing insects leaving the crop, and the two quadrants of each trap that were furthest away from the crop were designated as capturing insects entering the crop (Figure 1). The collecting tray of each trap was filled with 1 L of water containing detergent (to reduce the surface tension of the water and ensure efficient capture of insects) (Virkon S; Antec International Ltd., www.antec.com). The traps were left for five days, and then the insects were collected, and placed in labelled vials containing 70% ethanol.

In the laboratory, insects were sorted to taxonomic orders and species where possible. The capture rates of five key flower-visiting species were analyzed in more detail, as these species were found to dominate flower visitation in a pilot trial conducted one year prior to the present study (Laura Mesa, unpublished data). The five species were a non-native bumblebee, *Bombus terrestris* L (Hymenoptera: Apidae), a non-native honeybee, *Apis mellifera* L., a native solitary bee, *Lasioglossum sordidum* Smith (Halictidae), a non-native drone fly, *Eristalis tenax* L. (Diptera: Syrphidae), and a native dark hover fly, *Melangyna novaezealandiae* Macquart. All specimens were deposited at Plant and Food Research, Lincoln, New Zealand.

### Statistical analyses

For comparisons of the abundance of flowervisiting insects entering and leaving fields, only two quadrants within an individual trap inside the field could be designated as entering or leaving with a great deal of certainty (see above), whereas counts from traps outside the crop were made from all four trap quadrants of each trap. Therefore, it was necessary to standardize abundance per trap by multiplying counts by a factor of two for all traps inside the field. Contingency tests (2×2) were calculated to determine whether the frequency of insects entering versus leaving the field differed between traps inside and outside the fields. Relative differences are expressed as log response ratios, calculated as log_10_((number entering + 1) / (number leaving + 1)).

## Results

### Flowering phenology

**Figure 2.**
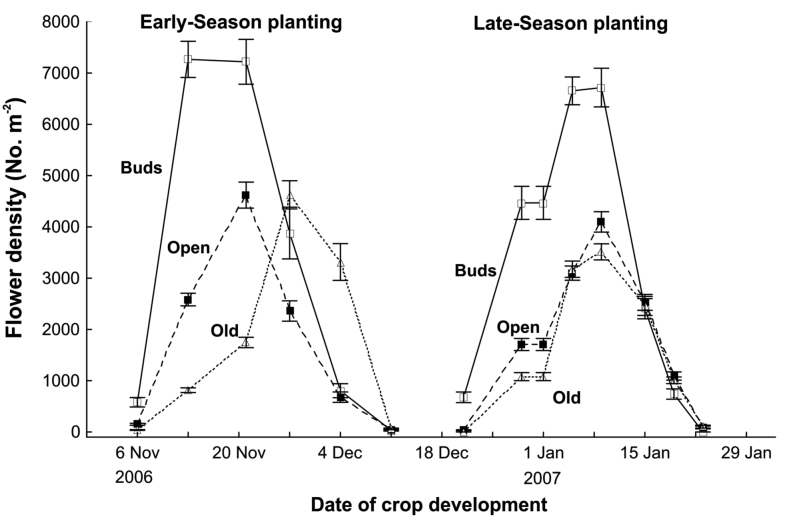
Variation in the mean (± 1 S.E.) densities of buds, open flowers, and old senesced *Brassica rapa* var. *chinensis* flowers during crop development, for both the spring-flowering crop (sown on 12 September 2006) and the summer-flowering crop (sown on 10 November 2006). High quality figures are available online.

The spring-flowering crop started flowering at day 48 (early November 2006), and flowering was completed by day 90, with the densities of buds, open flowers, and old flowers showing a broad overlap in occurrence, but distinct seasonal peaks (Figure 2). The summer-flowering crop started flowering substantially sooner, at day 38 (early December 2006), and the flowering period was much shorter, with a lower proportion of open flowers available at peak flowering (Figure 2).

### Window trap survey

**Figure 3.**
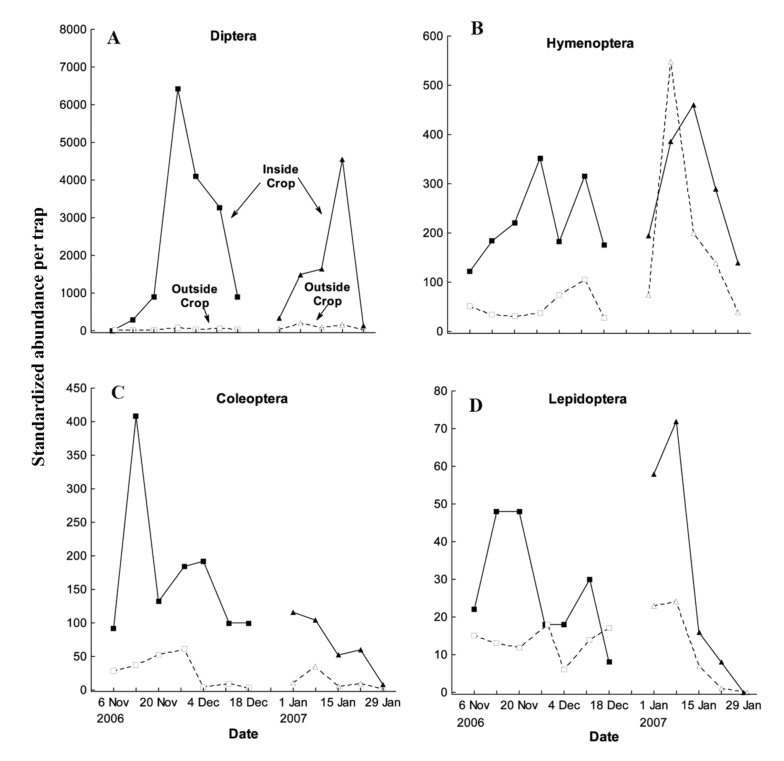
The four most abundant insect orders captured in window traps located inside (closed symbols and solid lines) and outside (open symbols and dashed lines) *Brassica rapa* var. *chinensis* crops in both spring-flowering and summer-flowering crops: a) Diptera, b) Hymenoptera, c) Coleoptera and d) Lepidoptera. Counts from traps inside the crop were only made from two of the four trap quadrants (in each of the four traps inside the field), whereas counts from traps outside the crop were made from all four trap quadrants of each of the four traps outside the field (see Materials and Methods). Therefore, standardized abundance per trap was calculated by multiplying counts by 2 for all traps inside the field. Overlapping data points offset for clarity. High quality figures are available online.

Six orders from the class Insecta were captured in window traps over the flowering period, with a total of 10,061 and 6654 specimens from the spring-flowering and summer-flowering crops, respectively. Diptera was the order with the highest number of specimens (12,823) and species (18) captured across 10 different families, with Stratiomyidae alone representing over 80% of the total capture rate (Table 1). Diptera abundance was greater in the spring-flowering crop (8213) than in the summer-flowering crop (4610), and was typically greater inside than outside the crop in both cases (Figure 3). Hymenoptera was the second most abundant order, with 2872 specimens and seven species comprised mainly of *A. mellifera* (1016 specimens), *L. sordidum* (969 specimens) and *B. terrestris* (552 specimens) (Table 1). Unlike Diptera, Hymenoptera had a greater abundance in the summer-flowering crop than in the spring-flowering crop, and Hymenoptera abundance was generally lower outside the crop than inside, with one notable exception at the peak of flowering in the late-season planting (Figure 3).

**Figure 4.**
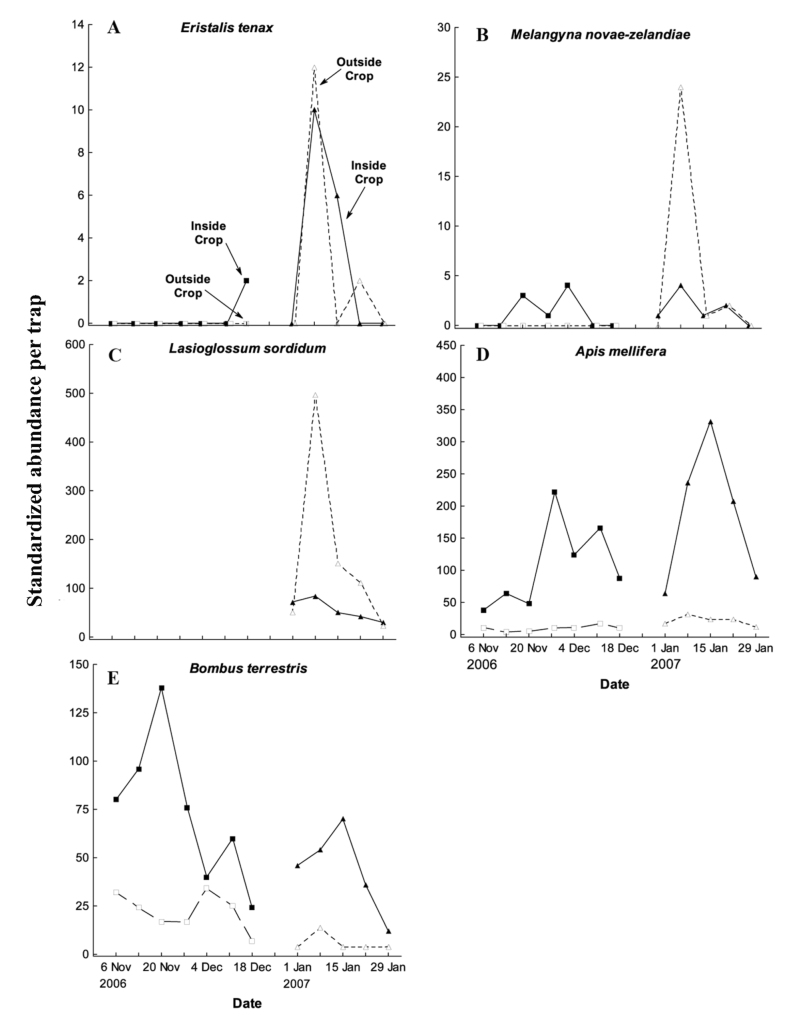
The five key flower-visiting species captured in window traps located inside (closed symbols and solid lines) and outside (open symbols and dashed lines) *Brassica rapa* var. *chinensis* crops in both spring-flowering and summer-flowering crops: a) *Eristalis tenax,* b) *Melangyna novaezelandiae*, c) *Lasioglossum sordidum* d) *Apis mellifera* and e) *Bombus terrestris*. See Figure 3 for further details. Overlapping data points offset for clarity. High quality figures are available online.

Five species identified as key pollinating species in previous studies (Rader et al. 2009; Howlett et al. 2011) accounted for 2603 specimens, dominated by *A. mellifera* (39%), *L. sordidum* (37%), and *B. terrestris* (21%), while *M. novaezealandiae* (2%) and *E. tenax* (1%) were comparatively less abundant. The most abundant species, *A. mellifera*, showed a consistent pattern of variation in abundance that tracked crop phenology in both the spring-flowering and summer-flowering crops (Figure 4). *A. mellifera* abundance was also consistently greater inside than outside the crop. These patterns were consistent with those observed for *B. terrestris* and (in the late-season crop only) for *L. sordidum*, with abundance tracking flowering phenology. However, it was surprising that the absolute frequency of *L. sordidum* outside the crop was substantially greater than inside the crop. *E. tenax* and *M. novaezealandiae* were comparatively rarely captured, and therefore trends in relative abundance were not possible to establish (Figure 4).

The overall frequency of insects entering versus leaving the field differed significantly between traps inside and outside the field for the summer-flowering crop (χ^2^ = 7.050, *p* = 0.008), but not for the spring-flowering crop (χ^2^ = 2.050, *p* = 0.152). Two species in the spring-flowering crop (*Megathereva bilineata* F. (Diptera: Therevidae) and *Scaptia adrel* Walker (Tabanidae)) and seven taxa in the summer-flowering crop (Tachinidae spp., *Delia platura* (Meigen) (Anthomyiidae), *E. tenax, Helophilus hochstetteri* Nowicki (Syrphidae), *Leioproctus* sp. (Hymenoptera: Colletidae), *Pieris rapae* L. (Lepidoptera: Pieridae), and moths) differed significantly in the frequency of insects entering versus leaving between traps inside and outside the crop (all χ^2^ > 4.29, *p* < 0.038; Table 1).

**Figure 5.**
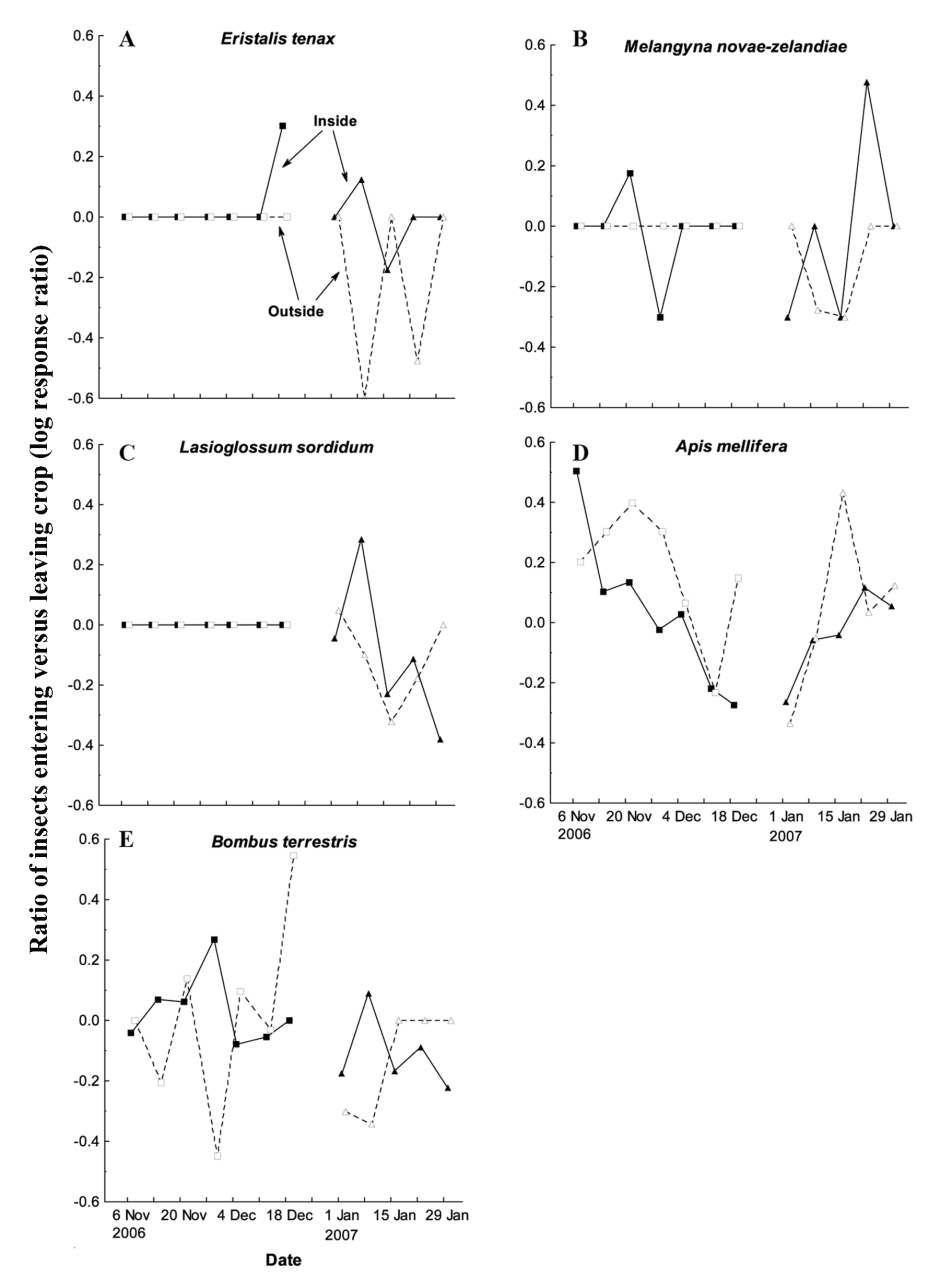
Ratio of abundance of five key flower-visiting species captured entering versus leaving *Brassica rapa* var. *chinensis* crops using window traps located both inside (closed symbols and solid lines) and outside (open symbols and dashed lines) the crop, in spring-flowering and summer-flowering crops. The log response ratio is calculated as log10((number entering + 1)/ (number leaving + 1)), and a value of +0.6 represents approximately four times as many individuals entering the crop as leaving, whereas a value of -0.6 represents approximately four times as many individuals leaving as entering the crop. High quality figures are available online.

For the five key flower-visiting species, the log response ratios of individuals entering versus leaving the crop varied considerably with crop phenology (Figure 5). With such wide variation, phenological trends in relative movement rates were difficult to establish for the two less common species, *E. tenax* (Figure 5a) and *M. novaezealandiae* (Figure 5b). For the more common species, log response ratios varied with season and stage of crop development. During the early stages of summer flowering, the capture rates of *L. sordidum* individuals entering the crop were one to two times greater than capture rates of individuals leaving the crop (Figure 5c). However, this ratio declined through time such that, by the end of the summer flowering period, two to three times more individuals were captured leaving the crop than entering (Figure 5c). This same trend was observed for *A. mellifera* in the spring-flowering crop, with over three times as many individuals entering versus leaving the crop in the early stages of flowering, but two to three times as many individuals leaving rather than entering the crop in the late stages of flowering (Figure 5d). Surprisingly, this trend was reversed for *A. mellifera* in the summer-flowering crop, with a comparatively greater numbers of individuals leaving rather than entering the crop in the early stages of flowering (Figure 5d). Finally, *B. terrestris* showed a somewhat idiosyncratic seasonal trend in capture rates, with an indication that log response ratios were highest at the peak of flowering, especially in the spring-flowering crop (Figure 5e).

## Discussion

Flower visiting insects play a vital role in the pollination of many flowering crops (Free 1993; Westerkampe and Gottsberger 2000; Klein et al. 2007). However, flower visitors may also contribute to undesirable gene movement among crop cultivars and wild relatives (Poppy and Wilkinson 2005; Funk et al. 2006). As different insect species are likely to contribute differentially to this process, it is essential to understand spatiotemporal variation in the distribution and movement patterns of flower-visiting insects during crop development. In this study, there were substantial differences in the diversity, species composition, and relative abundances of flower visiting insects captured in spring-flowering versus summer-flowering crops, as well as substantial variation throughout the flowering development cycle within each crop. As expected, the activity rates of most flower-visiting insects tracked the absolute availability of floral resources. However, peak activity times varied between insect species within the same trapping location in the crop, as well as between different trapping locations inside versus outside crop fields for the same insect species. Most notably, for some flowervisiting species, the apparent dispersal trajectories of insects entering versus leaving crop fields varied significantly in relation to spatial location inside or outside the crop, and stage of flower development. These findings could have potential implications for gene movement within and between *Brassica* crop fields through insect-mediated pollen transport.

### Flower visitor abundance and crop phenology

The four most frequently captured orders in this study, Diptera, Hymenoptera, Coleoptera and Lepidoptera, were also those typically recorded in other studies as the most common flower visitors in mass-flowering *Brassica* crops (Sahli and Conner 2007; Howlett et al. 2009a; Rader et al. 2009). Surprisingly, combined abundances of these orders were greater in the spring-flowering crop, rather than in the summer-flowering period when insect abundances would normally be expected to be greatest in the cool, temperate region. The high spring-flowering capture rates were driven by greater numbers of Diptera (particularly Stratiomyidae) and Coleoptera, whereas Hymenoptera and Lepidoptera were more abundant overall in the summer-flowering crop. Although Stratiomyidae flies have previously been recognized as flower visitors (Lamborn and Ollerton 2000; Souza-Silva et al. 2001; Howlett et al. 2009a, 2011) and pollinators of *Brassica* crops (Howlett et al. 2011), their dominance in both spring- and summer-flowering crops was unexpected. In New Zealand, the stratiomyid *Odontomyia* sp. can be highly variable in their transfer of pollen to pak choi stigmas, with individuals depositing between zero and several hundred grains in a single visit (Howlett et al. 2011). Given the large numbers of *Odontomyia* recorded in this study, they are likely to play a significant role in absolute pollen transfer within crops. In addition, their ability to move distances of at least 400 m (Rader et al. 2011) indicates they are likely to be key contributors to long distance pollen flow from *Brassica* crops.

Of the five key flower-visiting species considered in this study, there was considerable seasonal variation in the relative abundance of species between spring- and summer-flowering crops. Capture rates of the honeybee *A. mellifera*, the two syrphid flies *E. tenax* and *M. novaezelandiae*, and most notably the native solitary bee *L. sordidum*, were all greater in the summer-flowering crop than in the spring-flowering crop, whereas capture rates of the bumblebee *B. terrestris* were greater in the spring-flowering crop. These gross seasonal patterns of variation among orders and species masked a more subtle trend in which capture rates inside the crop fields tended to be higher in spring- than summer-flowering crops, whereas the reverse was true outside the crop fields, where capture rates tended to be higher in the summerflowering crop. The dominant driver of these trends could be species-specific differences in seasonal activity patterns, dispersal capabilities, or behavioral aggregation in the crop, but the observed trends might also result from differences in relative resource availability in the surrounding landscape. In the case of *B. terrestris*, for example, the higher early-season activity may be due to a comparatively greater ability to tolerate cool climatic conditions compared with other species, or it may simply be that there are more mass-flowering crops available in the surrounding landscape in the summer period, and this availability has a dilution effect on the frequency of bumblebee foraging across numerous crops in the landscape. More generally, relative resource availability in the landscape needs to be considered in more detail before conclusions can be drawn about local determinants of seasonal pollinator activity.

As might be expected, peak capture rates of flower-visiting insects within each cropping season largely corresponded with the peak of the flowering period (Sihag and Khatkar 1999). Floral resource availability is well known to correlate with increased activity of flower visitors, both in cropping systems (Westphal et al. 2003) and natural ecosystems (Hegland and Boeke 2006). In our study, capture rates of all insect orders, as well as the five key flower-visiting species, clearly tracked floral resource availability inside crop fields, in both the spring-flowering and summer-flowering crops. Interestingly, capture rates of several flower-visiting species 50 m outside the crop fields also tracked floral resource availability inside the fields. This suggests that the mass-flowering crop had a strong landscape-level influence on insect activity rates beyond the boundaries of the crop itself. Whether the landscape influence occurs primarily through an aggregative response of adult insects already active in the surrounding area, or through increased reproductive output of local individuals, is open to question. For social Hymenoptera, such as *A. mellifera* and *B. terrestris*, it is most likely that more individual foragers were simply recruited to the crop as floral resource availability progressively increased. *Apis mellifera*, of course, will be heavily influenced by the distance of *Brassica* crops from commercial beehives (Sabbahi et al. 2005); in the present study, several managed *A. mellifera* hives were located within 2 km of both trial crops. *Bombus terrestris* colonies are not actively managed for *Brassica* pollination services in this region, but, again, forager recruitment is likely to be an aggregative phenomenon. Similarly, Diekötter et al. (2010) found that foraging bumblebee recruitment to mass-flowering crops when in peak flower reduced pollination services to other plants in the landscape that relied on bumblebees for pollination. Whether or not this same adult aggregative phenomenon applies to solitary flower visitors foraging for nectar or pollen is less certain. *Lassioglossum sordidum*, for example, is a small solitary bee, and is therefore likely to have a much more limited foraging range than the larger *A. mellifera* and *B. terrestris* (Gathmann and Tscharnke 2002), so the extreme peak in capture rates at the height of flowering might be due to an increase in reproductive output of individuals, and enhanced local population density in the immediate vicinity of the crop.

### Frequency of movement of flower visitors entering and leaving crops

If capture rates of flower-visiting insects in and around *Brassica* crops were determined primarily by patterns of aggregation in the landscape, then periods of peak activity in the crop might not correspond to periods of greatest risk of pollen transport between crops. Instead, greatest risk might arise from high differential immigration rates from other parts of the landscape into the crop field during the build-up phase of local aggregation, or from high differential emigration rates out of the crop post-aggregation. For key flower-visiting species, a comparison of the relative log response ratios of individuals entering versus leaving crops at different stages of the flowering cycle suggested that there were strongly non-random patterns of variation in insect movement in relation to crop phenology. Most notably for the honeybee, *A. mellifera*, which had high capture rates in both spring- and summer-flowering crops, and is known to carry relatively large pollen loads compared to many other flower visiting species (Howlett et al. 2011), the log response ratio shifted from strongly positive in the early stages of spring flowering (three times as many individuals entering compared to leaving the crop field) to strongly negative in the late stages of spring flowering (two to three times as many individuals leaving rather than entering the crop field). These remarkable differences in relative movement trajectories occurred at times when absolute capture rates were comparatively low in the crop. This suggests that landscape-wide pollen transport between crops might also increase at these times, outside of peak flowering, when local resource availability is relatively low. Naturally, without absolute measures of insect dispersal between crop fields, there can only be circumstantial inference made about the significance of these altered movement trajectories for landscape-wide pollen transport. Nevertheless, the data do provide strong evidence that relative movement rates within and between fields do vary with flowering phenology.

These seasonal trends in relative movement trajectories will depend to a great extent on the relative distribution and availability of other floral resources in the landscape, not just local flower density within the focal crop itself. If landscape-wide floral resource availability varied markedly from spring to summer, this may also explain why phenological variation in the movement trajectories of *A. mellifera* differed so strikingly between the spring-flowering and summer-flowering crop. Moreover, other key flower-visiting species, such as *L. sordidum*, had very different patterns of phenological variation in movement trajectories over the same time interval, potentially suggesting species-specific responses to local versus landscape-level resource availability.

The movement of insects between crops and the surrounding environment has important implications for unwanted pollen flow (between crops and weedy relatives). Despite high variation in relative capture rates, the data indicate strongly non-random patterns of variation in insect movement in relation to crop phenology, with early-season aggregation of flower-visiting insects entering and remaining in the crop, and then mass emigration of flower-visiting insects leaving the crop late in the flowering season. Although pollen movement late in the flowering cycle might contribute relatively little to total seed set (and hence crop production), extensive late-season pollinator redistribution in the landscape could contribute disproportionately to long-distance gene movement between crops at these times. This redistribution appears to be dependent on species-specific dispersal capabilities, and the relative resource availability in the wider landscape surrounding crop fields. A greater understanding of the relationships between changing flowering phenology and pollinator redistribution in the landscape is required to develop more accurate risk assessment models for insect-mediated gene movement.

**Table 1.**
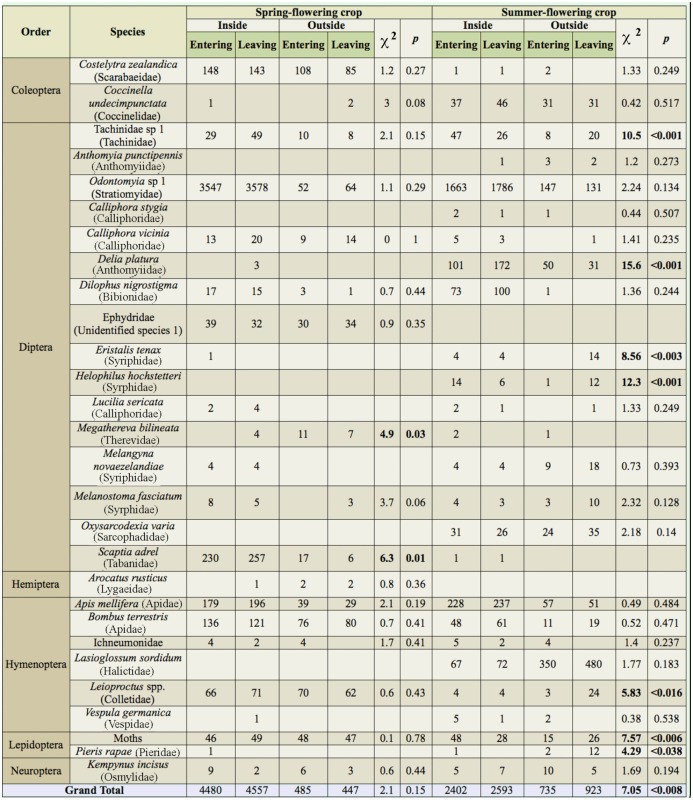
A comparison of the relative abundances of insects captured in directional window traps placed both inside and outside of a spring-flowering and a summer-flowering crop of *Brassica rapa* var. *chinensis* in Lincoln, Canterbury, New Zealand. The list includes both species known to visit flowers and transport pollen, as well as other species captured incidentally. Entering and Leaving indicate the direction of movement of the insects when they were captured in the window trap. For each species, in each of the two crops, a χ^2^ goodness of fit test was used to determine whether the frequency of individuals entering versus leaving the crop differed significantly between window traps placed inside and outside the crop. Bold *p*-values are significant at *p* < 0.05.
